# Concentration-dependent effects of tobacco smoke on airway inflammation and remodeling in asthmatic models

**DOI:** 10.3389/fimmu.2026.1775512

**Published:** 2026-03-10

**Authors:** Yinhe Feng, Yingting Peng, Tong Feng, Yubing Yue, Xiaolong Li, Ding Han, Chunfang Zeng

**Affiliations:** Department of Respiratory and Critical Care Medicine, Deyang People’s Hospital, Affiliated Hospital of Chengdu University of Traditional Chinese Medicine (TCM), Deyang, China

**Keywords:** airway inflammation, airway remodeling, asthma, inflammatory factors, tobacco smoke

## Abstract

**Objective:**

This study aimed to investigate the effects of different concentrations of tobacco smoke on airway remodeling and airway inflammation in mouse models of asthma with distinct inflammatory phenotypes.

**Methods:**

Asthmatic mouse models were established, including an eosinophilic asthma (EA) model via ovalbumin (OVA) sensitization/challenge, a neutrophilic asthma (NA) model via OVA combined with lipopolysaccharide (LPS, 10μg) sensitization/challenge, and a mixed granulocytic asthma (MGA) model via OVA combined with LPS (0.1μg) sensitization/challenge. These mice were then exposed to tobacco environments at concentrations of 100 mg/m³ and 800 mg/m³. Body weight changes and clinical symptoms were observed and recorded. Levels of inflammatory factors (TNF-α, IFN-γ, IL-6, IL-12, MCP-1, and IL-10) in bronchoalveolar lavage fluid (BALF) were measured by ELISA. Lung histopathological changes were assessed via hematoxylin and eosin (HE) staining, while bronchial goblet cell hyperplasia and mucus secretion were observed using periodic acid-Schiff (PAS) staining. Pulmonary fibrosis was assessed using Masson’s trichrome staining.

**Result:**

Compared with the blank control group, mice in the EA, NA, and MGA model groups showed significantly reduced body weight growth rate (P < 0.001) and aggravated respiratory symptoms. Tobacco smoke exposure further exacerbated these symptoms in a concentration-dependent manner. Regarding inflammatory factors, the levels of pro-inflammatory factors (TNF-α, IFN-γ, IL-6, MCP-1, IL-12) in BALF were significantly elevated, while the level of the anti-inflammatory factor IL-10 was significantly decreased (P < 0.001) in the EA, NA, and MGA model groups. Tobacco smoke exposure significantly aggravated the airway inflammatory response in these different asthmatic models in a concentration-dependent manner. HE staining results demonstrated that tobacco smoke exposure worsened pathological injuries in the lung tissues of the different asthmatic models, including alveolar collapse, inflammatory cell infiltration, and epithelial cell necrosis, with the most severe damage observed in the 800 mg/m³ exposure group. PAS staining results revealed a significant increase in the percentage of goblet cells in the lung tissues of the different asthmatic models exposed to 800 mg/m³ tobacco smoke (P < 0.01, P < 0.05, and P < 0.01, respectively). Masson staining results showed that the degree of pulmonary fibrosis in mice from the EA model group, NA model group, and MGA model group exposed to an 800 mg/m³ tobacco environment was significantly increased (P < 0.001, P < 0.001, and P < 0.01, respectively).

**Conclusion:**

Tobacco smoke exposure exacerbates clinical symptoms, airway inflammation (by up-regulating pro-inflammatory factors and down-regulating the anti-inflammatory factor IL-10), and lung pathological damage in asthmatic mice in a concentration-dependent manner.

## 1 Introduction

Bronchial asthma is a heterogeneous disease characterized by chronic airway inflammation and airway hyperresponsiveness, affecting more than 300 million people worldwide with a continuously rising prevalence (1). The classic type 2 (Th2-high) inflammatory pathway, marked by eosinophilic infiltration, elevated IgE, and overproduction of cytokines such as IL-4, IL-5, and IL-13, represents the dominant immunopathological mechanism in most mild-to-moderate allergic asthma cases (2). However, approximately 20%–40% of patients with severe or refractory asthma exhibit non-type 2 or mixed inflammatory phenotypes, in which the airways are predominantly infiltrated by neutrophils or co-dominated by both eosinophils and neutrophils, accompanied by markedly elevated Th1/Th17-associated cytokines (e.g., IFN-γ, IL-12, and IL-17) (3–5). These distinct inflammatory endotypes differ significantly in glucocorticoid responsiveness, exacerbation frequency, and long-term prognosis, making precise identification of asthma inflammatory phenotypes a critical focus of current clinical and translational and basic research.

In addition to endogenous immune dysregulation, environmental exposures play a pivotal role in the initiation and exacerbation of asthma. Tobacco smoke is one of the most well-established modifiable risk factors. Both active and passive smoking substantially increase asthma incidence, trigger acute exacerbations, reduce corticosteroid sensitivity, and accelerate irreversible decline in lung function (6). Tobacco smoke contains over 7,000 chemical compounds that directly impair the airway epithelial barrier, activate proinflammatory signaling pathways such as NF-κB and MAPK, induce massive release of proinflammatory cytokines (TNF-α, IL-6, IL-8, MCP-1), and simultaneously suppress the anti-inflammatory cytokine IL-10, resulting in sustained amplification of airway inflammation (7). More importantly, tobacco smoke can promote the phenotypic shift from Th2- to Th1/Th17-dominant responses by upregulating transcription factors such as T-bet and RORγt, thereby exacerbating non-eosinophilic inflammation (8). Animal studies have further demonstrated that tobacco smoke exposure significantly enhances goblet cell metaplasia, mucus hypersecretion, subepithelial collagen deposition, and airway smooth muscle hyperplasia, ultimately driving irreversible airway remodeling (9).

To date, most studies investigating the effects of tobacco smoke on asthma have relied exclusively on the classic ovalbumin (OVA)-sensitized/challenged eosinophilic asthma model, whereas systematic comparative analyses of tobacco smoke exposure in neutrophilic and mixed granulocytic severe asthma models remain scarce (10). Therefore, in the present study, we established a neutrophilic asthma (NA) model and a mixed granulocytic asthma (MGA) model by intranasal administration of high-dose (10 μg) and low-dose (0.1 μg) lipopolysaccharide (LPS), respectively, on the basis of the conventional eosinophilic asthma (EA) model. Mice were subsequently exposed to two concentrations of tobacco smoke (100 mg/m³ and 800 mg/m³) to simulate mild and heavy tobacco exposure environments. By comprehensively evaluating clinical manifestations, inflammatory cytokine profiles, airway inflammatory cell infiltration, goblet cell metaplasia, and pulmonary fibrosis across multiple dimensions, we aimed to elucidate the impact of tobacco smoke exposure on airway inflammation and remodeling in asthma models with distinct inflammatory phenotypes, as well as its potential concentration-dependent characteristics, thereby providing experimental evidence for precision intervention strategies in severe clinical asthma.

## 2 Experimental materials

### 2.1 Experimental animals

Sixty specific pathogen-free (SPF) female BALB/c mice, aged 4 weeks and weighing 15 ± 2 g, were supplied by Chengdu Dossy Experimental Animal Co., Ltd. All animal experiments were conducted in the animal facility of Sichuan Lilai Biotechnology Co., Ltd. The laboratory animal use license number is SYXK (Sichuan) 2021-0246. The facility operates under barrier system conditions, with temperature maintained at 20–26 °C, relative humidity 40%–60%, and a 12 h light/12 h dark cycle. Mice were housed 3–4 per cage and fed SPF-grade maintenance chow provided by Chengdu Dossy Experimental Animal Co., Ltd. (production license No. SCXK (Sichuan) 2019-028).

This study was reviewed and approved by the Institutional Animal Care and Use Ethics Committee. The experimental design and protocol fully comply with the principles of safety and animal welfare and meet all relevant national regulations on the use of laboratory animals in medical research. The project was approved to proceed as planned (Animal Ethics Approval No. LLSN-2025117).

### 2.2 Main reagents

The primary reagents used in this study are listed in **Table 1**.

**Table 1:** Main reagents used in the experiment.

Reagent	Catalog no.	Manufacturer
Ovalbumin (OVA)	A5503	Sigma
Lipopolysaccharide (LPS)	ST1470	Beyotime Biotechnology
0.9% Normal Saline	L24010318	Sichuan Kelun Pharmaceutical Co., Ltd.
Formalin fixative	13400401	Xilong Scientific
Aluminum potassium sulfate	A7210	Sigma
Sodium dihydrogen phosphate	1010580101700	Tianjin Fuchen Chemical Reagents Co., Ltd.
Disodium hydrogen phosphate	1010590101700	Tianjin Fuchen Chemical Reagents Co., Ltd.
Formaldehyde (AR)	1340040101602	Sichuan Xilong Scientific Co., Ltd.
Absolute ethanol (AR)	100092680	Sinopharm Chemical Reagent Co., Ltd.
Dewaxing/Clearing agent	240131	Wuxi Jiangyuan Industrial & Trade Co.
Hematoxylin staining solution	H9627	Sigma-Aldrich
Eosin staining solution	YE2080	Hefei Bomei Biotechnology Co., Ltd.
Hydrochloric acid (AR)	7647-01-0	Chengdu Kelong Chemicals Co., Ltd.
Neutral balsam	1004160	Sinopharm Chemical Reagent Co., Ltd.
Modified Masson’s trichrome stain	G1006	Servicebio, Wuhan
PAS staining kit	G1280	Solarbio, Beijing
Wright-Giemsa stain	G1020	Solarbio, Beijing
Mouse TNF-α ELISA Kit	ZC-39024	Shanghai ZC Biotechnology Co., Ltd.
Mouse IFN-γ ELISA Kit	ZC-37905	Shanghai ZC Biotechnology Co., Ltd.
Mouse IL-6 ELISA Kit	ZC-37988	Shanghai ZC Biotechnology Co., Ltd.
Mouse IL-10 ELISA Kit	ZC-37962	Shanghai ZC Biotechnology Co., Ltd.
Mouse IL-12 ELISA Kit	ZC-37966	Shanghai ZC Biotechnology Co., Ltd.
Mouse MCP-1 ELISA Kit	ZC-38075	Shanghai ZC Biotechnology Co., Ltd.

### 2.3 Main instruments and consumables

Instruments and consumables used are listed in **Table 2**.

**Table 2:** Main experimental instruments and consumables.

Item	Model	Manufacturer
Electronic balance	FA1004N	Shaoxing Super Instruments Co., Ltd.
Compressed-air nebulizer	403T	Jiangsu Yuyue Medical Equipment Co., Ltd.
Rotary microtome	Leica-2016	Leica, Germany
Automatic tissue dehydrator	JT-12S	Wuhan Junjie Electronic Co., Ltd.
Tissue embedding machine	BMJ-A	Changzhou Zhongwei Electronic Instrument Factory
Tissue flotation bath	PHY-III	Changzhou Zhongwei Electronic Instruments Co., Ltd.
Digital slide scanner	SQS-600P	Shenzhen Shengqiang Technology Co., Ltd.
Microplate reader	SpectraMAX Plus384	Molecular Devices, USA
Digital water bath	HH-6	Shanghai Lichen Bangxi Instrument Technology Co.
Automated plate washer	PW-960	Shenzhen Huysong Technology Development Co., Ltd.
Ultra-pure water system	SSY-II	Sichuan Shui Siyuan Environmental Technology Co.
Pipettes (1000 μL/200 μL/50 μL)	—	Dragon Laboratory Instruments Co., Ltd.
Pipette tips (1000 μL/200 μL/50 μL)	—	Biosharp
Digital trinocular microscope	BA210Digital	Motic China Group Co., Ltd.

### 2.4 Establishment of asthmatic mouse models

After a 7-day acclimatization period, sixty 4-week-old female SPF BALB/c mice were ear-tagged, weighed, ranked by body weight.

Sixty specific pathogen-free (SPF) female BALB/c mice were randomly allocated into ten groups (n = 6 per group): blank control group, eosinophilic asthma (EA) model group, EA + 100 mg/m³ tobacco exposure group, EA + 800 mg/m³ tobacco exposure group, neutrophilic asthma (NA) model group, NA + 100 mg/m³ tobacco exposure group, NA + 800 mg/m³ tobacco exposure group, mixed granulocytic asthma (MGA) model group, MGA + 100 mg/m³ tobacco exposure group, and MGA + 800 mg/m³ tobacco exposure group.

#### 2.4.1 Model establishment

After acclimatization, each group received the following interventions:

Blank control group: No special treatment; mice were maintained under normal conditions.

EA model: On days 0, 7, and 14, mice were intraperitoneally sensitized with 0.2 mL of ovalbumin (OVA) solution (100 μg OVA + 1 mg aluminum potassium sulfate dissolved in PBS). Starting on day 21, mice were placed in a transparent plastic chamber (24.0 cm × 15.5 cm × 8.5 cm) and challenged by aerosolized 6% OVA (dissolved in PBS) for 30 min once daily at 09:00. After 7 consecutive days, model validation was performed (**Supplementary Figure 1**).

NA model: Sensitization was identical to the EA group. Starting on day 21, mice underwent the same OVA aerosol challenge protocol as the EA group. Additionally, each challenge was preceded by intranasal administration of 10 μg LPS 30 minutes before OVA aerosol challenge on days 2, 4, and 6 of the challenge period (**Supplementary Figure 2**).

MGA model: Sensitization was identical to the EA group. OVA challenge protocol was the same as above, but each challenge was preceded by intranasal administration of 0.1 μg LPS 30 minutes before OVA aerosol challenge on days 2, 4, and 6 of the challenge period (**Supplementary Figure 3**).

##### 2.4.1.1 Tobacco exposure intervention

100 mg/m³ tobacco environment: Starting from day 1 (and from day 21 onward, 30 min after OVA nebulization), mice were exposed to tobacco smoke at a total particulate matter concentration of 100 mg/m³ (generated from 1 commercial cigarette with filter removed) for 30 min per session, twice daily (8 h apart), 5 days/week, for 4 consecutive weeks.

800 mg/m³ tobacco environment: Identical schedule, but using smoke generated from 5 commercial cigarettes (filters removed) to achieve 800 mg/m³ total particulate matter concentration.

The total particulate matter (TPM) concentration in the exposure chamber was monitored gravimetrically during each session by drawing chamber air through pre-weighed glass fiber filters at a known flow rate; post-exposure filter mass gain divided by sampled air volume yielded the TPM concentration (mg/m³), which was maintained at the target levels of 100 mg/m³ or 800 mg/m³.

Tobacco smoke was generated from a commercially available filtered cigarette brand widely used in China (Hongtashan, Yunnan Tobacco Co., Ltd., China; tar yield 10 mg/cigarette, nicotine 0.8 mg/cigarette, as labeled). Filters were removed prior to smoke generation to standardize exposure and simulate unfiltered mainstream smoke characteristics.

### 2.5 Observation of general clinical features

From the start of the experiment, the general condition of mice in each group was observed and recorded daily at a fixed time, including mental state, activity level, coat luster, fecal consistency, and urination. Body weight of all animals was measured upon arrival and weekly throughout the experiment.

### 2.6 Animal euthanasia and sample collection

Mice were euthanized 24 h after the final OVA aerosol challenge. Animals were anesthetized by intraperitoneal injection of sodium pentobarbital (50 mg/kg). Loss of consciousness was confirmed, pain reflex was absent, and no response was observed upon toe pinch with forceps. Cervical dislocation was then performed, and cessation of heartbeat and respiration was verified.

Post-euthanasia dissection and sampling procedures were as follows:

Bronchoalveolar lavage fluid (BALF): In 6 mice per group, the neck and chest skin was incised, tissues were separated layer by layer to fully expose the trachea. A transverse incision was made in the upper trachea, a 21G needle was inserted for tracheal intubation, and the right lung was ligated. Using a syringe, 0.3 mL ice-cold PBS was slowly instilled into the left lung and gently withdrawn. This procedure was repeated three times; total recovery rate was approximately 60%. Recovered fluid was filtered to remove mucus, immediately centrifuged (2000 r·min−1, 4 °C, 10 min), supernatant was stored at –80 °C, and the cell pellet was resuspended in PBS for Wright–Giemsa staining to classify inflammatory cells.

Right upper lung lobe: In 6 mice per group, fixed in 10% neutral buffered formalin for subsequent histopathological examination.

### 2.7 Hematoxylin and eosin staining

Fixed tissues were dehydrated in an automatic tissue processor (75% ethanol 2 h, 85% ethanol 1 h, 95% ethanol 1 h, 100% ethanol I–IV each 20 min, clearing agent I 25 min, clearing agent II 30 min, paraffin I 30 min, paraffin II–III each 1 h), embedded, and sectioned. Sections were then processed as follows:

(1) Dewaxing and rehydration: clearing agent I 30 min, clearing agent II 30 min, absolute ethanol I & II 5 min each, 95%–75% ethanol 5 min each, running tap water 5 min. (2) Hematoxylin staining 5–10 min, rinse in running tap water until clear. (3) Differentiation in acid-alcohol ~3 s, rinse. (4) Bluing in weakly alkaline solution, rinse. (5) Alcohol-soluble eosin 3 min. (6) Gradient ethanol dehydration, clearing, mounting with neutral balsam.

Images were acquired using a digital microscope imaging system. Each slide was first examined at low magnification, followed by capture of images at 200× and 400×. All lung tissues underwent gross and histopathological examination. Lesions were semi-quantitatively scored according to severity.Non-gradable lesions (e.g., cysts) were recorded as present/absent.

### 2.8 Periodic Acid–Schiff staining

Paraffin sections were processed as in section 2.4 up to the cutting stage, then:

(1) Dewaxing and rehydration (same as HE). (2) Rinse in tap water and distilled water. (3) Oxidation in PAS oxidizer 5–10 min. (4) Schiff reagent 10–20 min. (5) Running tap water 10 min. (6) Hematoxylin counterstain 1–3 min. (7) Acid differentiation, rinse. (8) Bluing, rinse 3 min. (9) Gradient ethanol dehydration, clearing, mounting.

Images were acquired as described. Two 400× images of terminal or respiratory bronchioles per section were captured. Goblet cells and bronchial epithelial cells were manually counted. Goblet cell percentage = (number of goblet cells/number of epithelial cells) × 100.

### 2.9 Masson’s trichrome staining

Paraffin sections were processed as in section 2.4, then:

(1) Dewaxing and rehydration (same as HE). (2) Overnight incubation in potassium dichromate. (3) Heating at 63 °C for 1 h. (4) Ponceau-fuchsin solution 10 min, brief distilled water rinse. (5) Phosphomolybdic acid treatment until collagen fades (seconds to 2 min). (6) Aniline blue ~2 min until collagen is blue. (7) Gradient ethanol dehydration, clearing, mounting.

Two 400× images per section were analyzed using Image-Pro Plus 6.0 software (Media Cybernetics, USA) to measure collagen fiber area. Collagen percentage = (collagen area/total field area) × 100.

### 2.10 Wright–Giemsa staining of BALF smears

(1) BALF cell smears were air-dried. (2) 2–3 drops of Wright–Giemsa stain were added to cover the smear for 1–2 min. (3) Equal volume of 0.01 M phosphate buffer (pH 6.4–6.8) was added, mixed gently, and stained for 3–5 min. (4) Gentle rinse with running water. (5) Air-dried or blotted dry, mounted.

Images were acquired at 100× and 400×. Differential cell counts were performed under oil immersion.

Macrophages: 10–30 μm, irregular or round, eccentric nucleus, abundant pale-to-deep blue cytoplasm containing fine gray-red or purple granules. Neutrophils: larger than erythrocytes, multilobed nucleus, pale purple-red cytoplasm. Eosinophils: Bilobed nucleus, abundant bright orange-red cytoplasmic granules that are uniformly sized and refractile. Lymphocytes: Small to medium size (slightly larger than erythrocytes), round dense nucleus occupying most of the cell volume, thin rim of pale blue cytoplasm without prominent granules.

### 2.11 Enzyme-linked immunosorbent assay

The following protocol was used for all cytokine kits:

(1) Kits were equilibrated to room temperature for 30 min; required strips were removed. (2) Standards (50 μL of each concentration) were added to standard wells. (3) Samples (50 μL) were added to sample wells; blank wells received no sample. (4) Except blank wells, 100 μL HRP-conjugated detection antibody was added to each well; plates were sealed and incubated at 37 °C for 60 min. (5) Wells were washed 5 times with wash buffer (1 min soak each). (6) 50 μL each of substrates A and B were added; incubated at 37 °C in the dark for 15 min. (7) 50 μL stop solution was added; OD was read at 450 nm within 10 min.

### 2.12 Statistical analysis

Data were analyzed using SPSS 20.0 software and expressed as mean ± SD. Inter-group comparisons were performed by one-way analysis of variance (One-Way ANOVA). When the ANOVA indicated a significant difference (P < 0.05), pairwise comparisons were conducted using Tukey’s Honestly Significant Difference (HSD) *post-hoc* test to control for multiple comparisons. P < 0.05 was considered statistically significant. Graphs were generated using GraphPad Prism 8.0.

## 3 Results

### 3.1 Effects of tobacco concentration on the general condition of mice with different inflammatory asthma phenotypes

During the modeling period, the general condition of mice was observed. Mice in the blank control group exhibited normal behavior, steady breathing, glossy fur, and no obvious coughing. In contrast, mice in the EA model group, NA model group, and MGA model group showed tachypnea, nasal discharge, dull and lackluster fur, and a significantly increased frequency of coughing compared with the blank control group. Mice in both the 100 mg/m³ and 800 mg/m³ tobacco exposure groups displayed a clearly noticeable increase in coughing frequency compared with their respective non-exposed asthmatic model groups, with the 800 mg/m³ tobacco exposure group showing an even more pronounced increase in coughing frequency than the 100 mg/m³ group. The above manifestations were consistent with the clinical symptoms of asthma.

**Figure 1:**
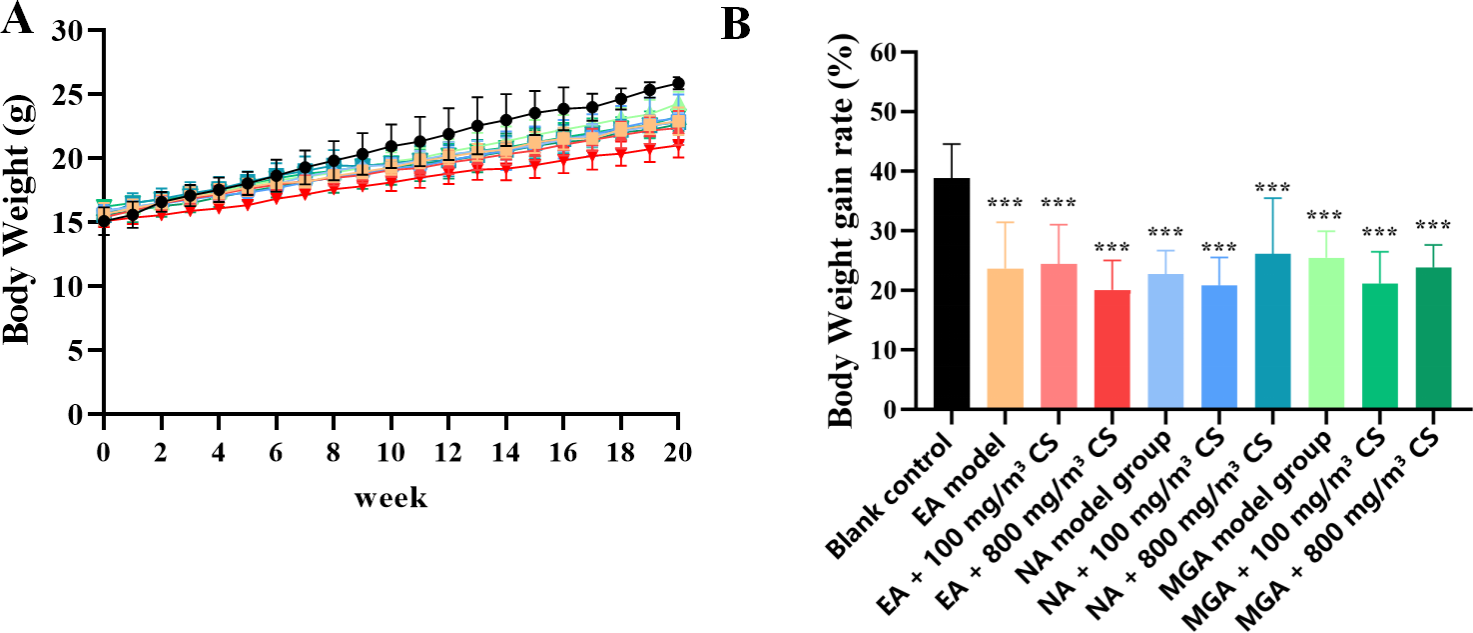
Body weight changes in mice of each group during the modeling period. **(A)** Body weight changes of mice in each group during the modelingperiod; **(B)** Body weight gain rate of mice in each group before and after modeling; compared with the blank control group, *** indicates P < 0.001. CS, Cigarette smoke.

Body weight of mice in each group was recorded weekly during the modeling period. As shown in **Figure 1**, the average body weight gain rate before and after modeling in the EA model group (46.84% ± 11.35%), EA + 100 mg/m³ tobacco group (45.82% ± 12.45%), EA + 800 mg/m³ tobacco group (39.32% ± 6.36%), NA model group (44.41% ± 4.56%), NA + 100 mg/m³ tobacco group (45.49% ± 9.44%), NA + 800 mg/m³ tobacco group (47.22% ± 9.33%), MGA model group (54.32% ± 6.30%), MGA + 100 mg/m³ tobacco group (43.75% ± 3.37%), and MGA + 800 mg/m³ tobacco group (45.89% ± 8.37%) was significantly lower than that in the blank control group (72.08% ± 11.06%) (P < 0.001). These results indicate that asthma symptoms are closely associated with the systemic health status of mice.

### 3.2 Effects of tobacco concentration on inflammatory cytokine expression in mice with different inflammatory asthma phenotypes

To investigate the effect of tobacco concentration on inflammatory cytokine expression in mice with different inflammatory asthma phenotypes, BALF was collected from each group to measure the levels of pro-inflammatory cytokines (TNF-α, IFN-γ, IL-6, IL-12, MCP-1) and the anti-inflammatory cytokine IL-10.

**Figure 2:**
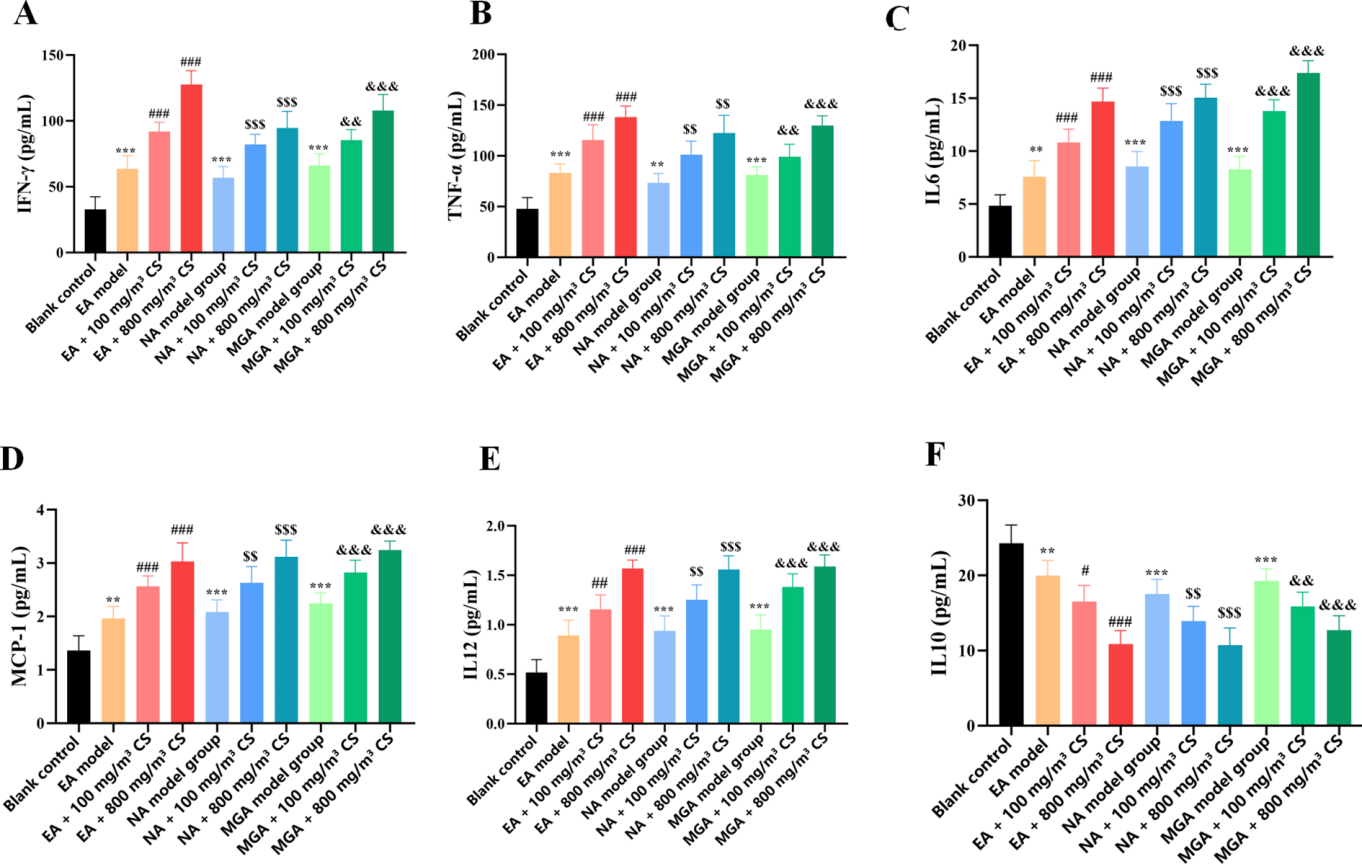
Effects of tobacco concentration on inflammatory cytokine expression in BALF of mice with different inflammatory asthma phenotypes. **(A)** TNF-α; **(B)** IFN-γ; **(C)** IL-6; **(D)** MCP-1; **(E)** IL-12; **(F)** IL-10. Compared with blank control group: *P < 0.05, P < 0.01, *P < 0.001; compared with EA modelgroup: #P < 0.05, ##P < 0.01, ###P < 0.001; compared with NA model group: $P < 0.05, $$ P < 0.01, $$$P < 0.001; compared with MGA modelgroup: &P < 0.05, &&P < 0.01, &&&P < 0.001. CS, Cigarette smoke.

Results showed that, compared with the blank control group, the levels of TNF-α (P < 0.001, P < 0.01, and P < 0.001), IFN-γ (P < 0.001, P < 0.001, and P < 0.001), IL-6 (P < 0.01, P < 0.001, and P < 0.001), MCP-1 (P < 0.01, P < 0.001, and P < 0.001), and IL-12 (P < 0.001, P < 0.001, and P < 0.001) in BALF were significantly elevated in the EA, NA, and MGA model groups (**Figures 2A–E**), whereas IL-10 levels were significantly reduced (P < 0.01, P < 0.001, and P < 0.001) (**Figure 2F**).

Compared with the respective model groups (EA, NA, MGA), both 100 mg/m³ and 800 mg/m³ tobacco exposure significantly increased the levels of TNF-α, IFN-γ, IL-6, MCP-1, and IL-12 while significantly decreasing IL-10 levels, with more pronounced effects generally observed at 800 mg/m³ (detailed P-values in **Figures 2A–F**). There were no significant differences in TNF-α, IFN-γ, IL-6, IL-10, IL-12, or MCP-1 levels among the EA, NA, and MGA model groups alone.

These data indicate that tobacco exposure exacerbates airway inflammation in different inflammatory asthma models in a concentration-dependent manner, characterized by increased pro-inflammatory cytokine expression and decreased anti-inflammatory cytokine expression. Notably, the significant elevation of IFN-γ and IL-12 (Th1-related cytokines) suggests that tobacco smoke may shift Th2-dominant allergic asthma toward a mixed Th1/Th2 phenotype. The increases in TNF-α, IL-6, and MCP-1, along with the decrease in IL-10, further indicate that tobacco smoke aggravates asthma symptoms by amplifying airway inflammatory responses.

### 3.3 Effects of tobacco concentration on inflammatory cell counts in mice with different inflammatory asthma phenotypes

**Figure 3:**
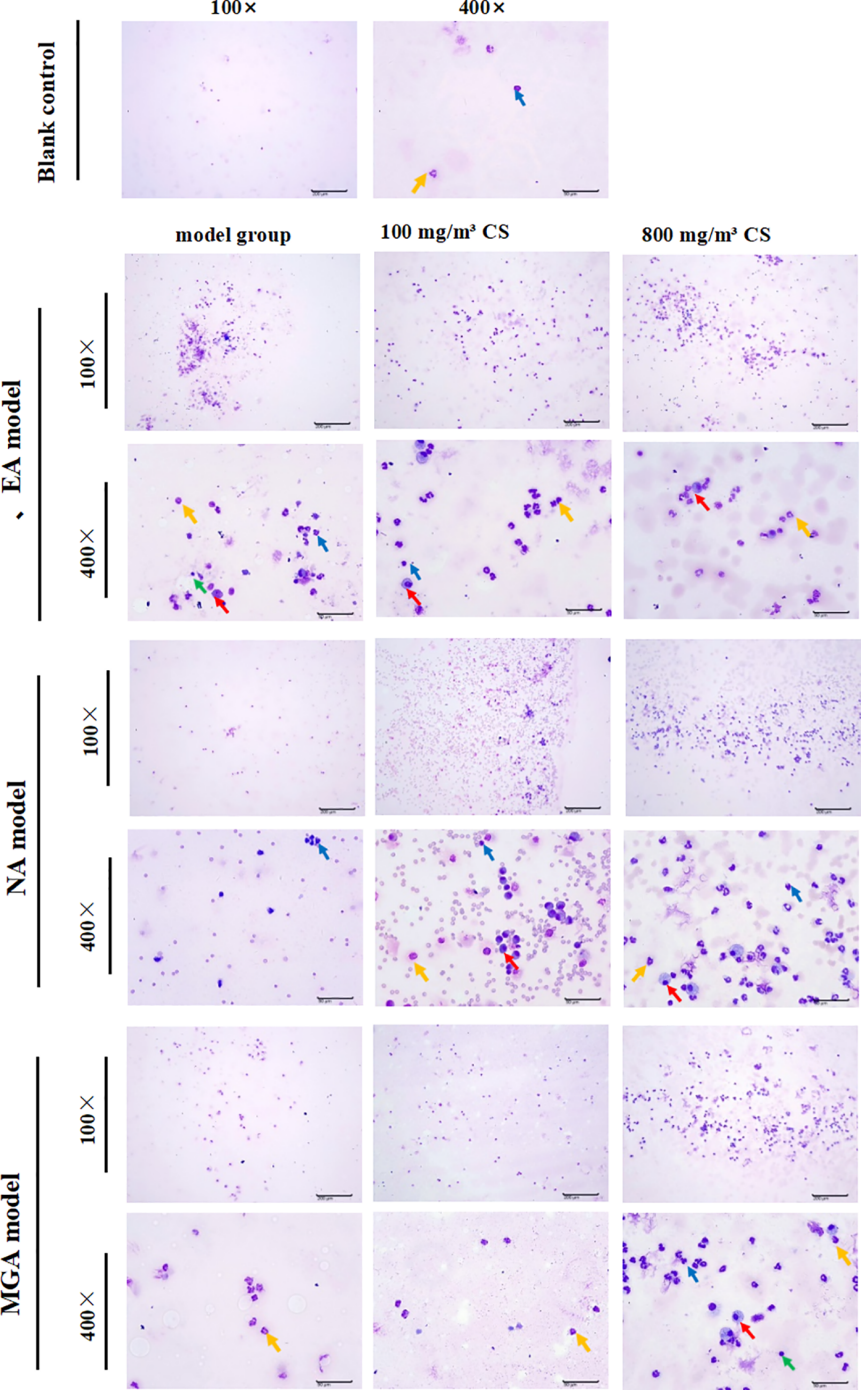
Wright-Giemsa staining showing the effect of tobacco concentration on inflammatory cells in BALF of mice with different inflammatory asthmaphenotypes. Lymphocytes (green); Neutrophils (blue); Eosinophils (yellow); Macrophages (red); Scale bar = 200 mm (100×), 50 mm (400×). CS, Cigarette smoke.

**Figure 4:**
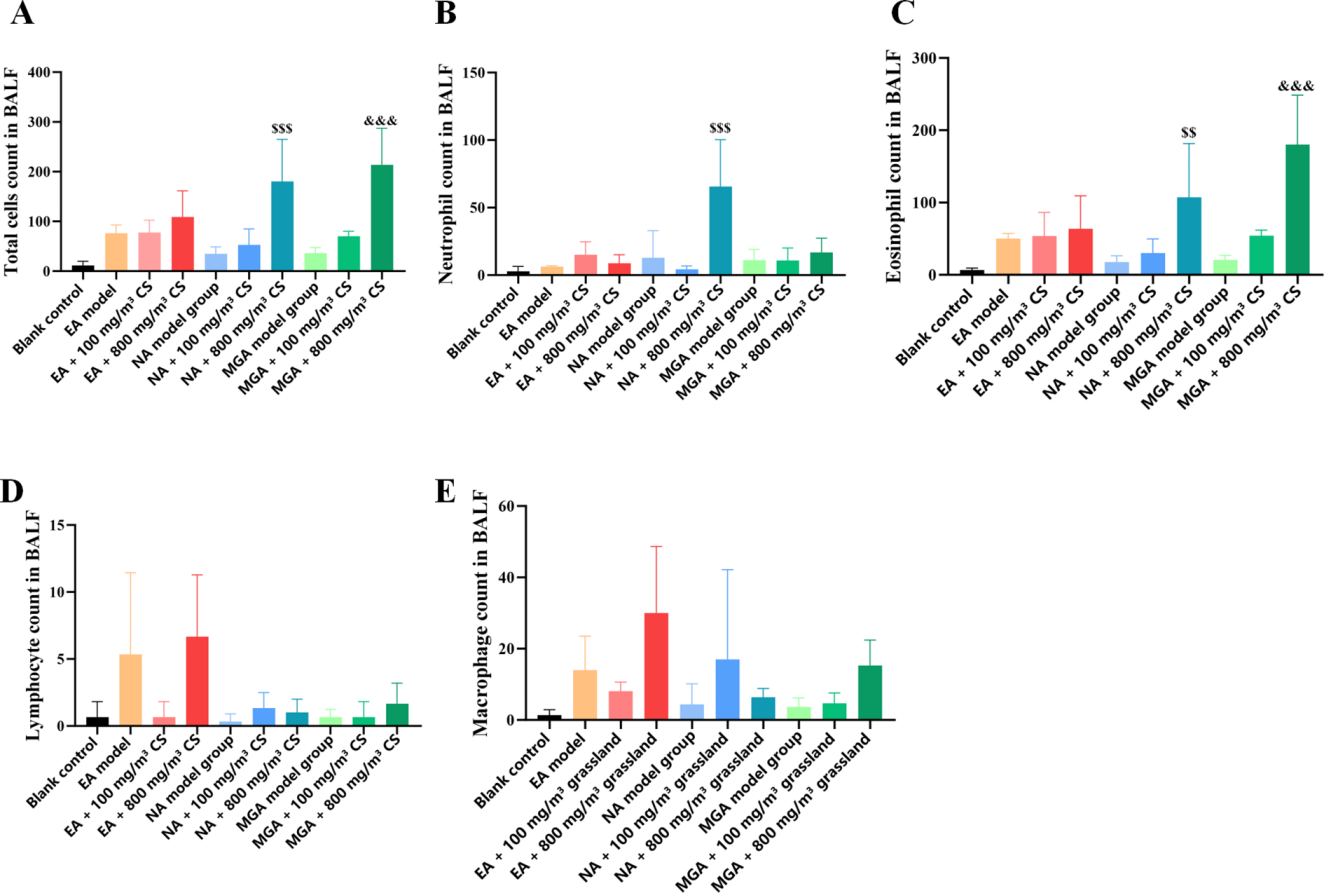
Statistical analysis of inflammatory cell counts in BALF of mice with different inflammatory asthma phenotypes. **(A)** Total cell count; **(B)** Neutrophils; **(C)** Eosinophils; **(D)** Macrophages; **(E)** Lymphocytes. Statistical symbols as in **Figure 2**. CS, Cigarette smoke.

Inflammatory cells in BALF resuspended pellets were observed and counted using Wright-Giemsa staining (**Figures 3**, **4**). Compared with the blank control group, total cell count, neutrophils, eosinophils, lymphocytes, and macrophages were increased in the EA, NA, and MGA model groups, but the differences were not statistically significant. Similar non-significant increases were observed in the 100 mg/m³ tobacco exposure groups compared with their respective model groups.

However, compared with the 800 mg/m³ tobacco exposure groups: Total cell count, neutrophils, and eosinophils were significantly higher than in the NA model group (P < 0.001, P < 0.001, and P < 0.01, respectively). Total cell count and eosinophils were significantly higher than in the MGA model group (P < 0.001 and P < 0.01, respectively).

These results suggest that tobacco exposure aggravates asthmatic responses in mice by promoting inflammatory cell infiltration, with the pro-inflammatory effect being more pronounced at 800 mg/m³.

### 3.4 Effects of tobacco concentration on lung histopathology in mice with different inflammatory asthma phenotypes

**Figure 5:**
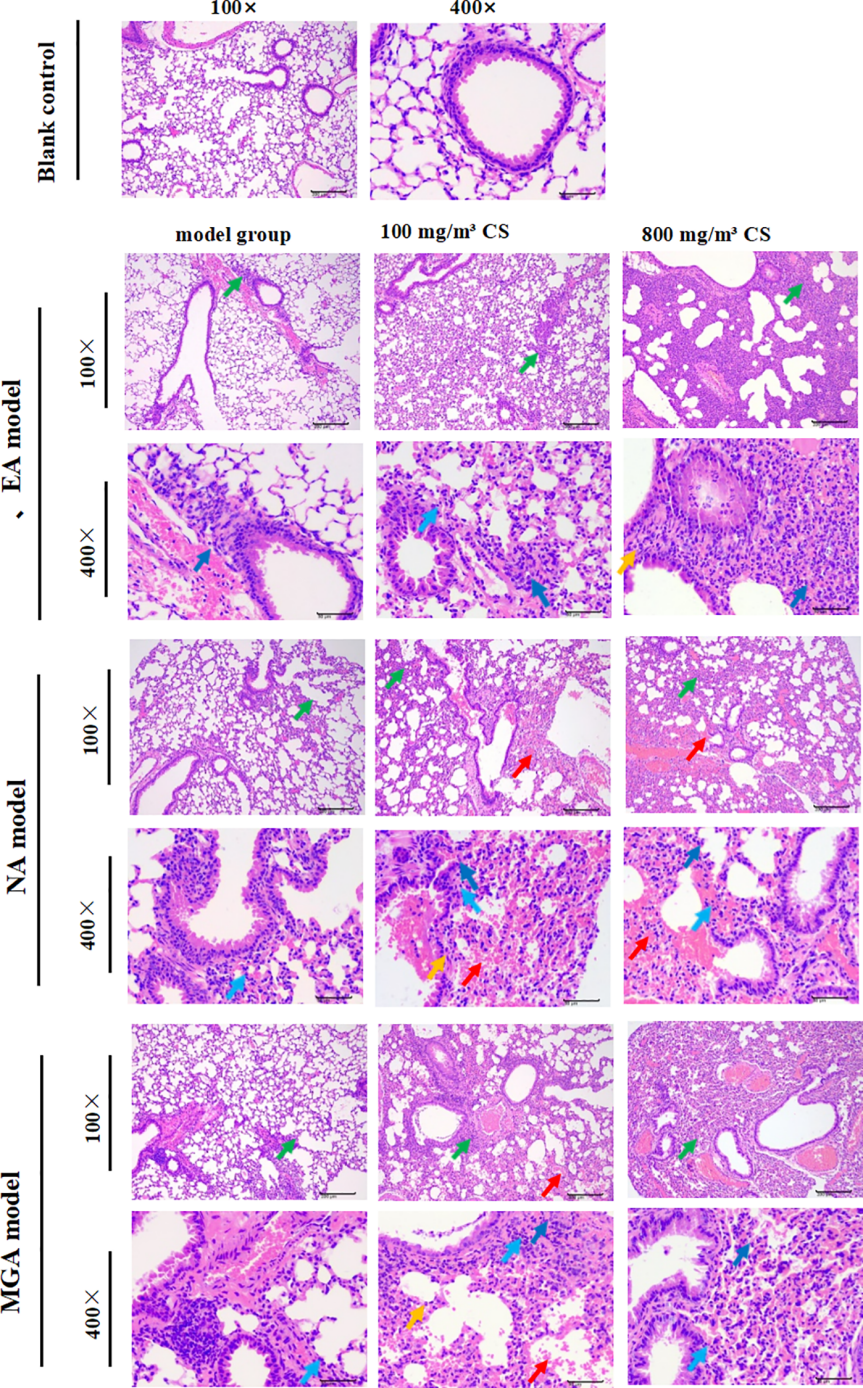
H&E staining showing the effect of tobacco concentration on lung histopathological changes in mice with different inflammatory asthmaphenotypes. Inflammatory cell infiltration (green); Neutrophils (dark blue); Mononuclear cells (light blue); Eosinophils (yellow); Hemorrhage (red);Scale bar = 200 mm (100×), 50 mm (400×). CS, Cigarette smoke.

A hallmark of asthma is airway obstruction associated with narrowing of the airway lumen, primarily caused by chronic airway wall inflammation accompanied by infiltration of eosinophils, neutrophils, and other inflammatory cells. The experiment evaluated histopathological changes in lung tissue using H&E staining (**Figure 5**). The blank control group showed normal bronchial epithelial morphology with no inflammatory cell infiltration or hemorrhage. The EA, NA, and MGA model groups exhibited mild focal (majority) to multifocal (minority) inflammatory cell infiltration, mainly around bronchioles, terminal bronchioles, and perivascular areas, consisting primarily of neutrophils, eosinophils, or mononuclear cells.

The 100 mg/m³ tobacco exposure groups showed mild focal to multifocal inflammatory infiltration and occasional hemorrhage. The 800 mg/m³ tobacco exposure groups displayed mild-to-moderate multifocal (majority) to diffuse (minority) acute or subacute inflammatory infiltration and hemorrhage, with inflammatory cells distributed within alveolar spaces and bronchial lumens.

Compared with the blank control group, histopathological lesions were more severe in all model groups. Compared with their respective model groups, both tobacco exposure groups (especially 800 mg/m³) exhibited more severe lung pathological damage. These data indicate that tobacco exposure exacerbates lung histopathological injury in different inflammatory asthma models in a concentration-dependent manner.

**Figure 6:**
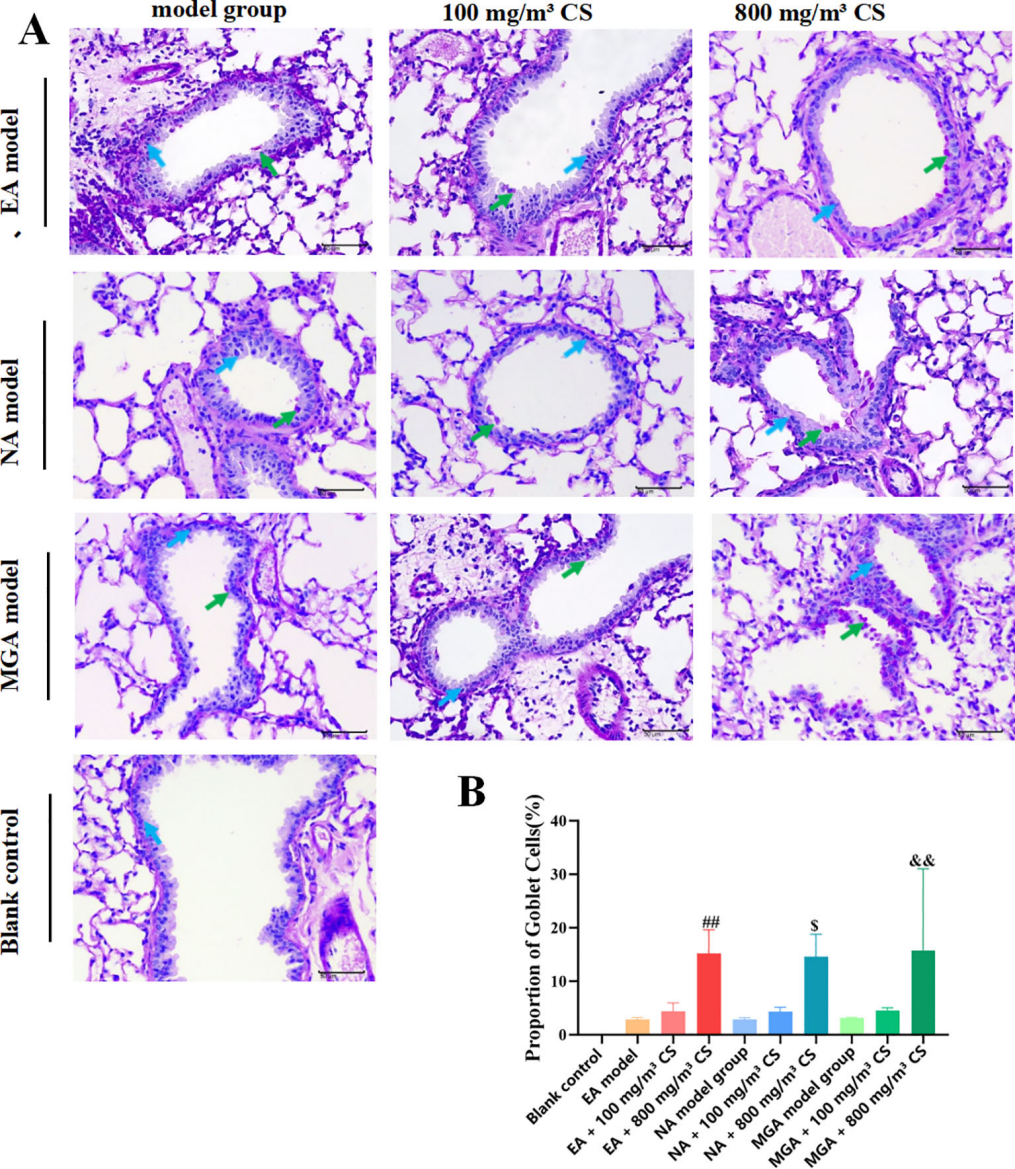
FPAS staining to observe the effect of tobacco concentration on goblet cell hyperplasia in the bronchial epithelium of mice with differentinflammatory asthma models. **(A)** PAS staining results; **(B)** Statistical results of the percentage (%) of goblet cells in the epithelium of terminalbronchioles/respiratory bronchioles; Goblet cells (green); Mucosal epithelial cells (blue); Scale bar = 50 mm (400×). CS, Cigarette smoke.

Goblet cell metaplasia (GCM) in airway epithelium is a hallmark of allergic asthma. PAS staining was used to assess goblet cell hyperplasia (**Figures 6A, B**). No goblet cell hyperplasia was observed in the blank control group. Compared with the model groups, the percentage of goblet cells increased non-significantly in the 100 mg/m³ tobacco groups but significantly in the 800 mg/m³ tobacco groups (P < 0.01, P < 0.05, and P < 0.01 across phenotypes). These results suggest that tobacco exposure promotes goblet cell hyperplasia, potentially exacerbating asthma by increasing mucus secretion.

**Figure 7:**
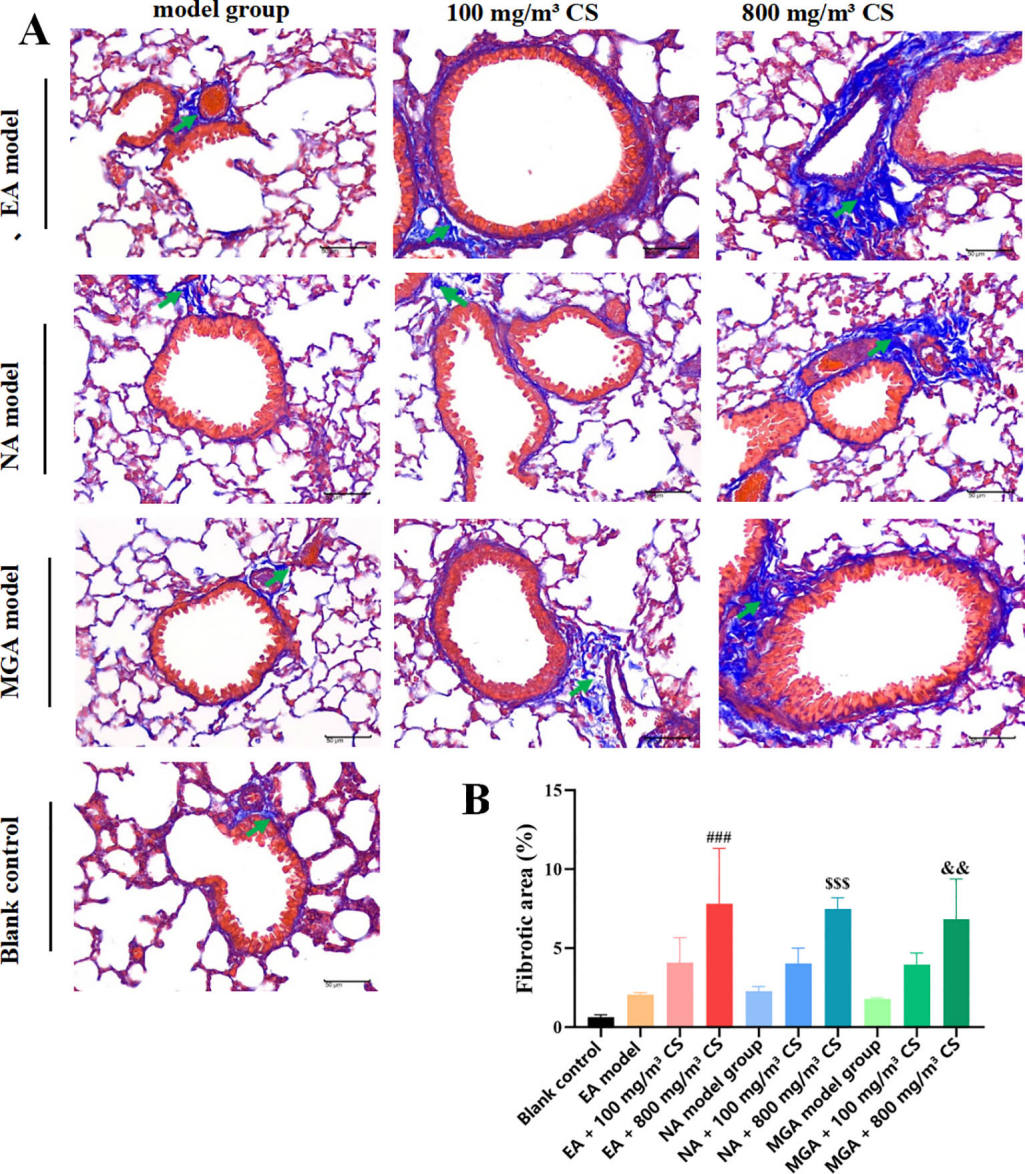
Masson trichrome staining showing the effect of tobacco concentration on pulmonary fibrosis and collagen deposition in mice with differentinflammatory asthma phenotypes. **(A)** Representative Masson trichrome staining images; **(B)** Statistical analysis of the percentage of fibrotic tissuearea (%) in lung tissue. Fibrotic tissue/collagen deposition (↑); scale bar = 50 mm (400×). Collagen fibers are stained blue, whereas muscle fibers,cytoplasm, and erythrocytes appear in varying shades of red. Compared with the blank control group: *P < 0.05, P < 0.01, *P < 0.001; comparedwith the EA model group: #P < 0.05, ##P < 0.01, ###P < 0.001; compared with the NA model group: $P < 0.05, $$ P < 0.01, $$$P < 0.001;compared with the MGA model group: &P < 0.05, &&P < 0.01, &&&P < 0.001. CS, Cigarette smoke.

Pulmonary fibrosis is considered a long-term, ultimately irreversible consequence of airway inflammation and remodeling in asthma. Masson trichrome staining was used to evaluate the degree of lung fibrosis (**Figures 7A, B**). Compared with the blank control group, fibrosis increased non-significantly in the model groups. The 100 mg/m³ tobacco groups showed a non-significant increase in fibrosis, whereas the 800 mg/m³ groups exhibited obvious fibrosis with significantly higher collagen expression percentages (P < 0.001, P < 0.001, and P < 0.01). These results indicate that tobacco exposure aggravates asthmatic responses in different inflammatory asthma models by promoting fibrotic processes.

## 4 Discussion

This study demonstrates that tobacco smoke aggravates airway inflammation and structural remodeling across EA, NA, and MGA asthma models, with greater effects at higher particulate concentrations. These findings align with prior mechanistic and *in vivo* data indicating that cigarette smoke amplifies airway cytokine production, mucus metaplasia, and extracellular matrix remodeling via oxidative and immune pathways.

First, our observation that proinflammatory cytokines TNF-α, IL-6, MCP-1, IFN-γ, and IL-12 rise while IL-10 declines in BALF after smoke exposure is consistent with tobacco smoke–driven activation of redox-sensitive transcriptional programs (e.g., NF-κB/AP-1) and epithelial injury that elevate TNF-α, IL-6, and chemokines and dampen anti-inflammatory tone, thereby intensifying airway inflammation (including neutrophil recruitment) (6). In mouse smoke-exposure models, increased BALF IL-6 and neutrophilia have been directly measured, supporting our cytokine and cellular readouts (11). Moreover, smoke enhances IFN-γ and modulates Th1/Th17 axes through IL-27/WSX-1 signaling, offering a mechanistic basis for the prominent IFN-γ and IL-12 increases we observed and a potential shift from Th2-dominant to mixed or Th1-skewed inflammation (12). Collectively, these pathways plausibly account for the concentration-dependent exacerbation we detected across phenotypes.

Second, histopathology corroborated the biochemical and cellular findings. We observed worsened epithelial damage, inflammatory infiltration, and hemorrhage on HE; heightened goblet cell metaplasia on PAS; and increased collagen deposition/fibrosis on Masson’s trichrome, most pronounced at 800 mg/m³. Prior smoke-model studies use identical stains and quantitative analyses to demonstrate goblet cell expansion and airway wall collagen accumulation, consistent with chronic remodeling (13). Mechanistic reviews further link smoke-derived oxidants to epithelial barrier dysfunction, mucous hypersecretion, MMP activation, and antiprotease inactivation—processes that promote goblet cell metaplasia and fibrotic matrix remodeling (6).

Third, the modeling strategy separating eosinophilic, neutrophilic, and mixed phenotypes using OVA with differential LPS co-exposure is supported by established mouse literature. Low-dose LPS with OVA yields mixed granulocytic responses with Th1/Th2/Th17 features, whereas higher-dose LPS drives neutrophilic, Th1/Th17-skewed inflammation and steroid insensitivity; H&E and PAS are standard for phenotype validation. The convergence of our smoke-induced increases in IFN-γ and IL-12 with these non-type 2 endotypes suggests that tobacco smoke may further entrench steroid-refractory pathways (14). This differential impact on non-Type 2 phenotypes is corroborated by recent reviews highlighting cigarette smoke’s role in promoting neutrophilic inflammation, airway remodeling, and steroid resistance in severe asthma (15).

Fourth, our finding of concentration dependence (100 vs 800 mg/m³) is directionally concordant with reports that the magnitude of smoke-induced inflammatory and tissue-damaging responses scales with particulate exposure and controlled delivery paradigms (nose-only vs whole-body) and with time/exposure-dependent increases in PAS-positive goblet cells and Masson-detectable collagen (16, 17). Mechanistic synthesis also emphasizes that higher oxidant burdens drive stronger cytokine outputs and matrix injury.

Mechanistic implications and relevance to severe, steroid-resistant asthma: Smoke-driven augmentation of IL-27/WSX-1 with resultant T-bet–dependent Th1 polarization, together with pathways fostering Th17 activity, provide a rationale for the increased IFN-γ and for mixed granulocytic inflammation in our models (18). LPS/TLR4-linked exacerbation and IL-27/IFN-γ signaling have been implicated in steroid-resistant airway hyperresponsiveness, aligning with the clinical challenge of smoke-exposed, non-eosinophilic asthma (5, 19). Structural remodeling observed here is congruent with smoke-induced activation of profibrotic growth factors and matrix-remodeling enzymes, offering potential targets but also indicating limited reversibility once fibrosis ensues. Furthermore, novel tobacco products such as heated tobacco products (HTPs) and e-cigarettes may pose similar risks, with recent data indicating increased asthma exacerbations, neutrophilic inflammation, and allergic responses, particularly in adolescents and severe phenotypes (20). Emerging evidence also links passive exposure to HTP aerosols with asthma attacks and persistent cough, underscoring the broader public health implications beyond traditional cigarettes (21).

Strengths include parallel assessment across EA, NA, and MGA phenotypes and multiparametric readouts (clinical, cytokines, histology). Limitations include the absence of direct lung function data and mechanistic inhibition (e.g., IL-27 blockade, MMP inhibition) to confirm causal nodes (22, 23). Nonetheless, concordance with established exposure models, histologic methods (HE/PAS/Masson), and immune mechanisms supports the robustness of our conclusions.

## 5 Conclusion

In summary, tobacco smoke exposure intensifies airway inflammation and remodeling across asthma phenotypes in a dose-dependent manner, offering experimental support for smoking cessation as a cornerstone of asthma therapy. These findings advocate for phenotype-specific strategies in managing smoke-exposed asthmatics, bridging basic research to clinical translation.

## Data Availability

The raw data supporting the conclusions of this article will be made available by the authors, without undue reservation.
