# Supplementary material for: Concentration-dependent effects of tobacco smoke on airway inflammation and remodeling in asthmatic models
Source: Front Immunol. 2026 Mar 10;17:1775512. doi: 10.3389/fimmu.2026.1775512 (PMC13008726; doi:10.3389/fimmu.2026.1775512)
Supplement: Supplementary file 1 [file DataSheet1.docx]

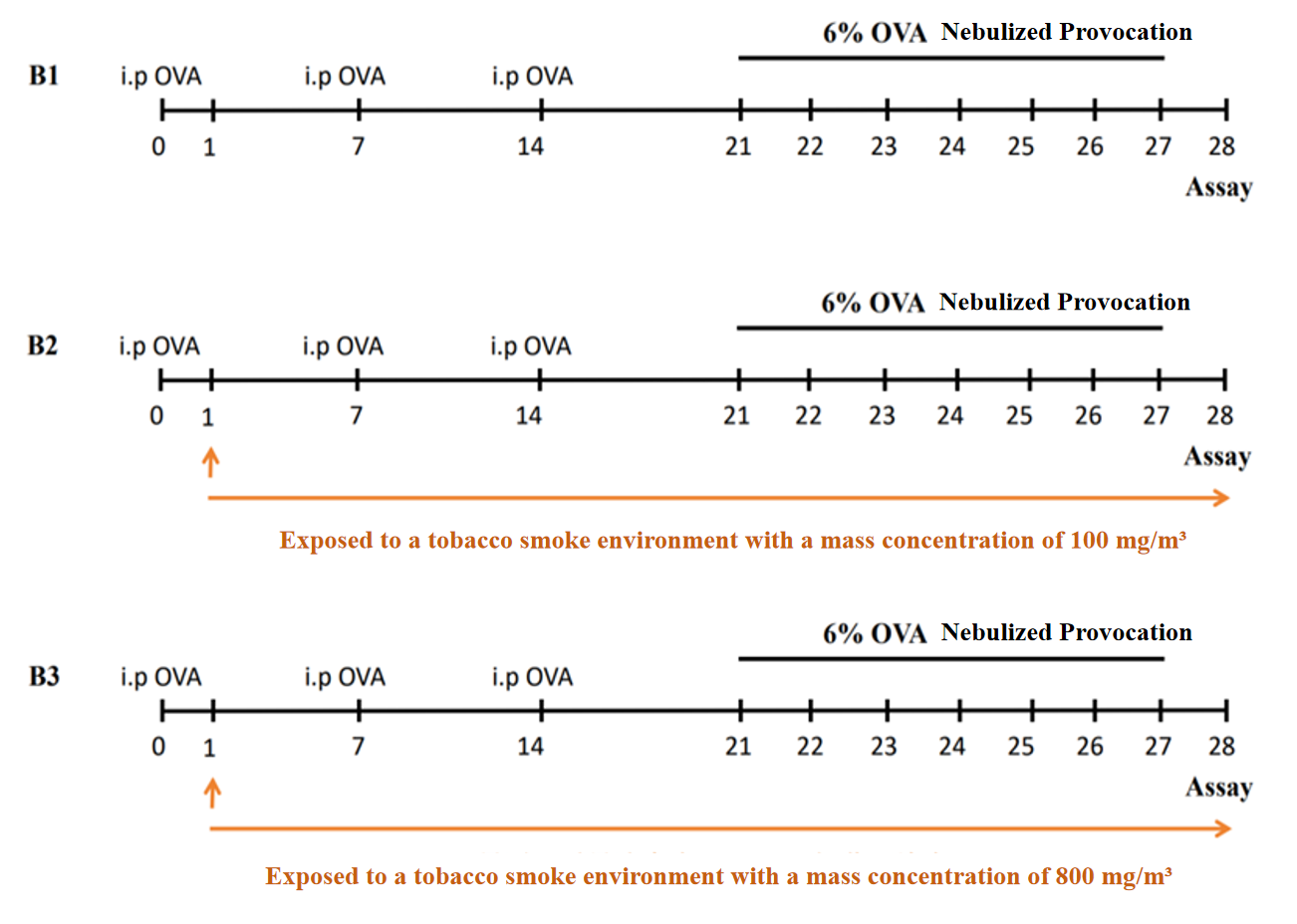


**Supplementary Figure 1. EA Group Experimental Flowchart**


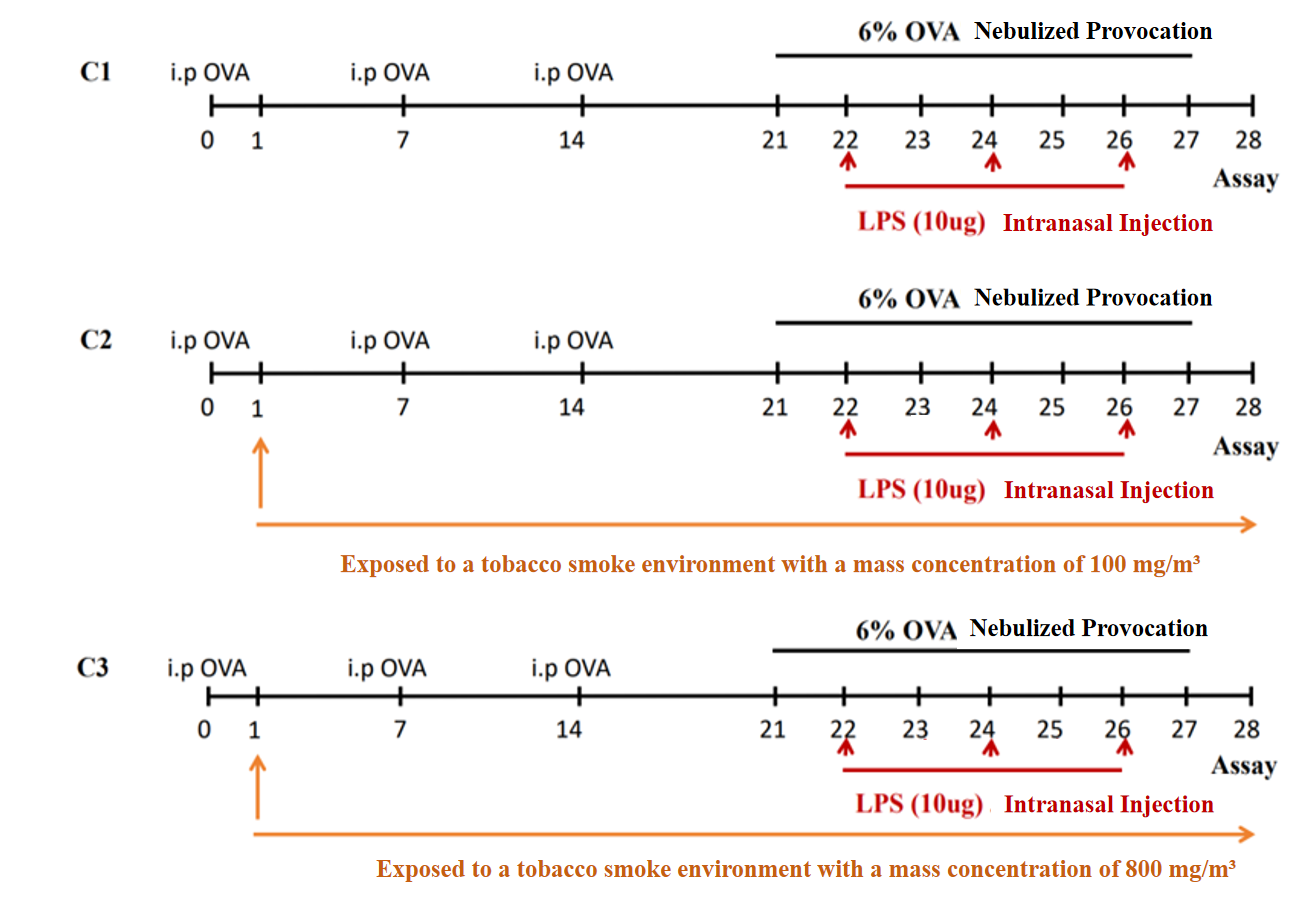


**Supplementary Figure 2. NA Group Experimental Flowchart**


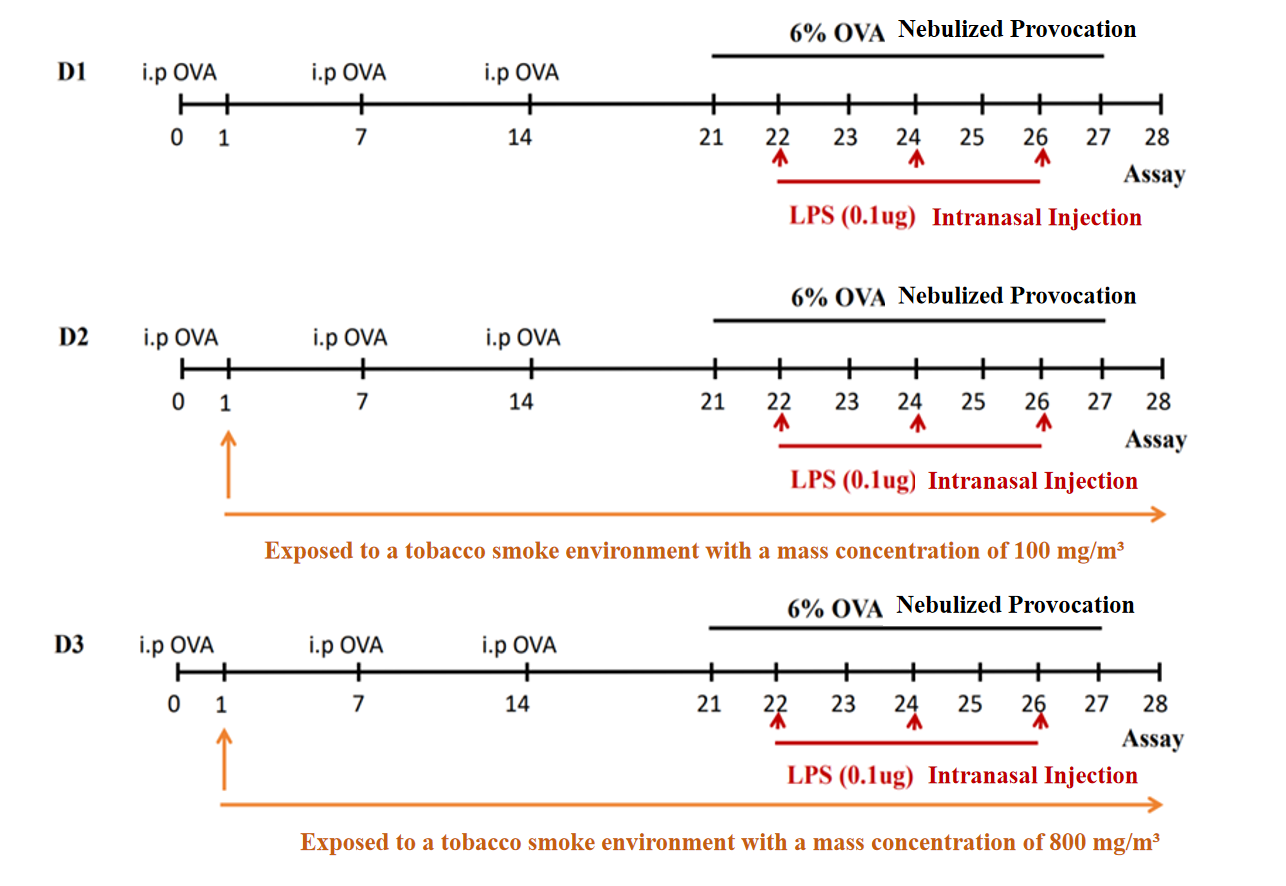


**Supplementary Figure 3. MGA Group Experimental Flowchart**
